# Molecular Characterization of Invasive *Neisseria meningitidis* Strains Isolated in Chile during 2010–2011

**DOI:** 10.1371/journal.pone.0066006

**Published:** 2013-06-11

**Authors:** Gisselle N. Barra, Pamela A. Araya, Jorge O. Fernandez, Jean-Marc Gabastou, Juan Carlos Hormazábal, Mabel Seoane, Paola C. Pidal, Maria T. Valenzuela, Ana Belén Ibarz-Pavón

**Affiliations:** 1 Sub-Department of Molecular Genetics, Institute of Public Health, Santiago, Chile; 2 Section of Bacteriology, Institute of Public Health, Santiago, Chile; 3 Pan American Health Organization, Washington, D.C., United States of America; 4 Biomedical laboratory department, Institute of Public Health, Santiago, Chile; Health Protection Agency, United Kingdom

## Abstract

**Background:**

With the upcoming licensure of Outer Membrane Protein-based vaccines against meningococcal disease, data on disease incidence and molecular characteristic of circulating *N. meningitidis* strains in Latin American countries is needed. Chile is, to date, one of the few countries in the region that has performed this type of work in a comprehensive collection of disease-associated strains from two consecutive years, 2010–2011.

**Methods:**

A total of 119 *N. meningitidis* strains isolated from patients with invasive disease in Chile in 2010–2011 were characterized by the National Reference Laboratory. Serogroup determination, MLST and *porA* typing were performed.

**Results:**

Serogroup B was predominant in both study years, but W135 experienced a noticeable increase in 2011 compared to 2010. ST-11 complex, ST-41/44 complex ST-32 complex were the most prevalent among the isolates, and were strongly associated with serogroups W135 (ST-11 Complex) and B (ST-41/44 and ST-32 complexes). Likewise, the major *porA* types detected were strongly associated with these three clonal complexes: P1.5,2 was found exclusively among W135:ST-11 isolates, whereas P1.7, 2–3 was only detected in C:ST-11. ST-41/44 isolates mainly had P1.10-8, and ST-32 complex were associated with a P1.18-8 *porA*.

**Conclusions:**

Our data show disease-associated *N. meningitidis* circulating in Chile are similar to those found in other parts of the world. The increase on W135:ST-11 isolates observed in 2011 foretold the unusual epidemiological situation experienced in the country in 2012, and MLST data show that this strain is indistinguishable from the one linked to the global Hajj 2000-related outbreak that occurred in 2001. Finally, this work demonstrates the importance of maintaining a strong national surveillance program integrating clinical, epidemiological and laboratory data and incorporating gold standard diagnostic and characterization techniques that allow the data to be compared all over the world.

## Introduction

*Neisseria meningitidis*, the meningococcus, is an exclusively human Gram negative pathogen that is carried asymptomatically by 8–20% of the population and can cause invasive disease in the form of meningitis and septicemia [1]. Invasive meningococcal disease (IMD) generally develops rapidly and has an associated mortality approaching 10%. Approximately 20% of survivors suffer from neurological and disabling sequelae in spite of prompt antibiotic therapy [2], [3]. The disease is a major concern in public heath worldwide and can occur as sporadic cases, outbreaks, and large epidemics [4]. Disease epidemiology varies among continents and countries, and therefore maintaining a well-established and coordinated clinical and laboratory-based surveillance system is crucial for identifying disease trends and timely identification of outbreaks, as well as evaluating the impact of preventive measures, including vaccination strategies [5].

Phenotypic characterization of *N. meningitidis* includes serogroup determination and is usually complemented by antigen typing, which is based on the variable regions of outer membrane proteins (OMP) and determines the serotype (porB) and serosubtype (porA).

The development of molecular biology techniques has resulted on classic monoclonal antibodies-based serotyping being replaced by DNA sequencing. Also, notwithstanding the importance of microbiological and phenotypic characterization, MLST has become the “gold standard” for the characterization of *Neisseria meningitidis.* Both MLST and sequencing of OMP, namely *porA* and *fetA* as recommended by the European Meningococcal Disease Society (EMGM) [6], have been incorporated as part of the routine work by national reference laboratories of many European countries and in the United States, making data comparable among countries, facilitating the identification of global spread of hyperinvasive strains and allowing investigations into the complex structure of bacterial populations [7], [8], [9].

However, these types of data are very scarce in Latin American countries, making it difficult to understand the disease epidemiology and how it relates globally. More so, the implementation of preventive measures against meningococcal disease, and specially vaccine development, is becoming almost exclusively dependent on molecular characteristics of strain, so it is now more important than ever to collect sufficient information before considering the possibility of including one of the existing or upcoming vaccines into the National Vaccination Scheme [10], [11], [12], [13], [14], [15], [16].

Disease associated serogroups vary widely across continents. Serogroup A is associated mainly with large scale epidemic outbreaks in the Africa Meningitis Belt [5]. Serogroup B is the leading cause of disease in Australia and New Zealand, as well as in Europe. In the United States, serogroups B, C and more recently Y account for the majority of invasive disease [5]. Data on serogroup distribution in Latin America and Caribbean countries is scarce. However, those obtained through the SIREVA II network indicates that although serogroups B and C are the main cause of IMD in the region, serogroup Y and specially W135 have increased in recent years [17], [18], [19].

In Chile, IMD is a disease of compulsory notification and all clinical cases must be reported to the epidemiology department of Ministry of Health. *N. meningitidis* isolates from reported cases are sent to the reference laboratory at the *Instituto de Salud Pública* (ISP) in the capital, Santiago [17]. Disease trends have experienced a progressive decline in the country since 2001, with incidence rates rarely surpassing 0.5/100,000 and serogroup B being the first cause of disease, followed by C and W135 [20], [21].

Three quadrivalent polysaccharide conjugate vaccines that use different protein carriers and cover serogroups A, C, W135 and Y are currently available and have been proved to be an effective measure to control IMD caused by these serogroups [22]. However, with the exception of strain-specific vaccines developed as a response to long-standing outbreaks in Cuba, Norway and New Zealand, no effective vaccine is available against serogroup B disease. There are currently several vaccines, some of them in very advanced stages of development, that target different OMPs. These vaccines have been designed using those OMP variants that are more commonly found among hyperinvasive meningococci and have shown promising results against all meningococcal strains, including serogroup B [22]. As these vaccines are being developed on the basis of genetic data, the implementation of molecular characterization into routine laboratory surveillance is of paramount importance for decision-making bodies for an evidence-based decision on the implementation of preventive measures against the disease.

This study presents a comprehensive analysis of IMD causing strains in Chile during a two-year period, from 2010 to 2011, combining traditional microbiological characterization with molecular typing of strains, and will provide an insight into the characteristics of circulating meningococcal strains in the country, as well as allow data to be compared to that from other parts of the world.

## Materials and Methods

### Bacterial Isolates

**Figure 1:**
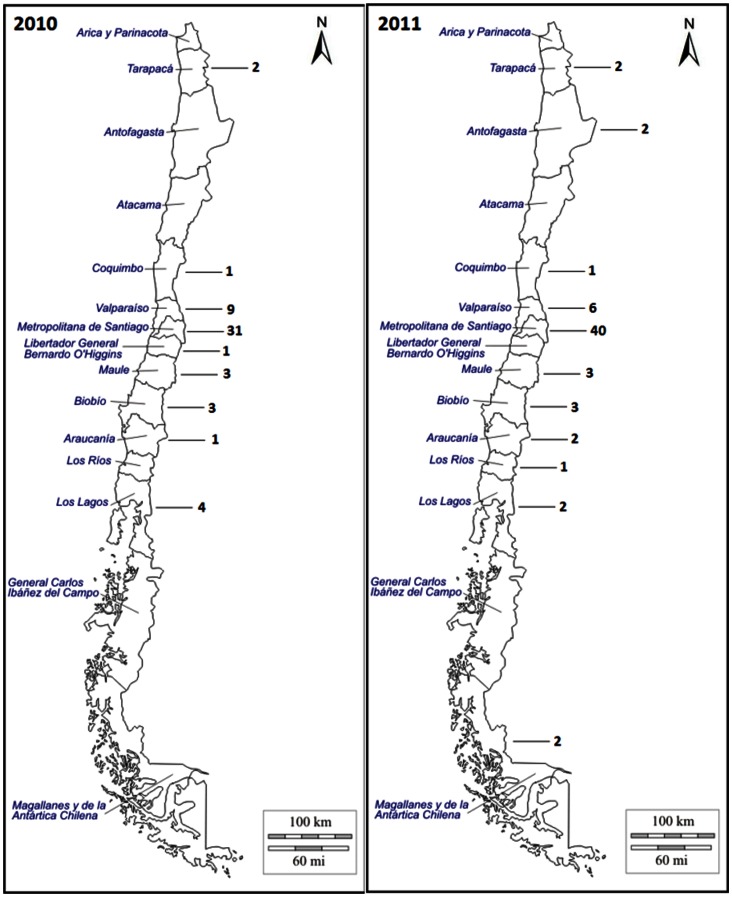
Detail of provenance of clinical isolates of Neisseria meningitidis collected during 2010 and 2011.

A total of 119 meningococcal strains isolated in 2010 (55 isolates) and 2011 (64 isolates) from an equal number of patients with IMD were included in this investigation. Isolates were received by the ISP as part of the routine disease surveillance and came from all over the country (Figure 1). Strain information was available from the laboratory report. Isolates were confirmed by standard microbiology techniques, and serogroup was determined by slide agglutination using commercial antiserum against meningococcal capsular polysaccharides (DIFCO, Beckton Dickinson).

### DNA Extraction

Isolates were retrieved from storage at −70°C and grown on chocolate agar plates for 16 hours at 37°C in a 5% CO_2_ atmosphere. About 5 colonies were suspended in 100 µl of molecular grade water and boiled for 10 minutes. Particulate matter was removed by sedimentation at 12,000×g, and the supernatant was used in subsequent analyses.

### Multilocus Sequence Typing (MLST) and porA Typing

MLST and *porA* typing were performed as previously described using the primers listed in the PubMLST website (http://pubmlst.org/neisseria/) [23], [24]. Sequencing reactions were performed using the BigDye terminator cycle sequencing kits (Applied Biosystems), and run in 3130 DNA sequencer (Genetic analyzer 3130, Applied Biosystems). Designation of Sequence Types (ST) and Clonal Complexes (CC) were retrieved by comparison on the PubMLST website. The strains for which the allelic profile query did not find an ST in the MLST database were submitted to the database curator for validation and assignment of ST numbers. *PorA* variable regions 1 and 2 (VR1 and VR2) were identified by querying the *porA* VR sequence database located at http://neisseria.org/nm/typing/pora/.

## Results

### Clinical Isolates

A total of 119 *N. meningitidis* strains were collected from Health Services located throughout Chile between 2010 and 2011 (Figure 1). Out of 119 isolates, 71 (59.6%) came from the Metropolitan region where the capital, Santiago, is located and houses over 40% of the country’s residents. Fifteen isolates (12.6%) came from region V, which corresponds to Valparaiso, a popular vacation and tourist resort. The rest of the isolates came from the rest of the country, and numbers ranged from six to one. Isolates were obtained mainly from blood (64, 53,7%) and CSF (52, 43,6%). The remaining isolates (2,5%) came from joint fluid (2 isolates, 1,6%) and amniotic fluid (1 isolate, 0,8%). Only one isolate *per* patient was processed. A total of 48 (40.3%) strains were isolated from patients presenting meningitis (defined as the presence of meningitis symptoms with or without sepsis), and 18 (15%) came from patients with septicemia. Diagnostic information was not recorded in the laboratory report for 53 isolates (44,5%). Patients were aged between one month and 90 years, and 66 strains (55.4%) were isolated from patients under five years. Isolates from patients aged 20 and older represented 27,7% of the total.

### Serogroup Distribution by Age-groups

Overall serogroup distribution and by age-group in 2010 and 2011 is shown in Table 1. Serogroup B was the most frequently detected, representing 63.6% and 51.6% in 2010 and 2011 respectively. The prevalence of serogroup C remained unchanged during the two-year period, with an average of 11.5%. A significant change on the prevalence of serogroup W135 was noticed, increasing from 7.8% in 2010 to 33.9% in 2011. This increase was specially noticed among those aged under the age of 5, and adults >20 years. In 2011 serogroup W135 became the most prevalent serogroup among isolates obtained from patients aged <1 year, representing 62.5% of all isolates from that age-group. On the contrary, serogroup B isolates obtained from patients <5 and >20 years of age showed the opposite trend.

**Table 1:** Serogroup distribution by age, 2010–2011.

	2010					2011				
Age	B	C*	W135*	Y	Other	B**	C	W135**	Y	Other
**<1**	7 (58,3%)	1 (8,3%)	3 (25%)	1 (8,3%)	0	6 (37,5%)	0	10 (62,5%)	0	0
**1–5**	13 (86,6%)	2 (13,3%)	0	0	0	16 (69,6%)	0	6 (26%)	1 (4,3%)	0
**6–14**	6 (85,7%)	1 (14,3%)	0	0	0	2 (66,6%)	1 (33,3%)	0	0	0
**15–20**	0	1 (50%)	0	1 (50%)	0	1 (50%)	1 (50%)	0	0	0
**>20**	9 (60%)	1 (6,6%)	1 (6,6%)	2 (13,3%)	2 (13,3%)	7 (38,8%)	5 (27,7%)	5 (27,7%)	1 (5,5%)	0
**Total**	35	6	4	4	2	32	7	21	2	0

**Note:**

* In 2010 two isolates serogroup C and two W135 not were include because age information was not available.

** In 2011 one serogroup B and one W135 isolates not were include because age information was not available.

### MLST Characterization

The 55 isolates collected in 2010 belonged to 25 different ST and could be grouped into 10 Clonal Complexes. ST-41/44 complex was the most prevalent with 16 (29.0%) isolates, of which 11 were ST-44, and four belonged to the newly described ST-9218 and ST-9233. All isolates from this CC were serogroup B. Twelve isolates belonged to the ST-32 complex and all of them were serogroup B. Two new STs, ST-9232 and ST-9235 belonging to this complex were detected. ST-11 complex was represented by 10 isolates, of which nine were ST-11, five W135:ST-11 and four C:ST-11. The remaining isolate from this complex was a newly described W135:ST-9222. Five additional new STs were detected among 2010 isolates, of which ST-9234 and ST-9226 belonged to ST-269 and ST-103 clonal complex respectively. The remaining three, ST-9221, ST-9219 and ST-9220 are yet to beassigned to a clonal complex. (Table 2).

**Table 2:** Genotypic characteristics of 55 isolates collected in 2010 causing meningococcal disease in Chile.

Number of isolates(N = 55)	ST	ST clonal complex	Serogroup	*porA* VR1	*porA* VR2
6	44	ST-41/44 complex/Lineage 3	B	19	13-1
1	44	ST-41/44 complex/Lineage 3	B	19	13
1	44	ST-41/44 complex/Lineage 3	B	19-12	13
1	44	ST-41/44 complex/Lineage 3	B	22	14-3
1	44	ST-41/44 complex/Lineage 3	B	21	4
1	44	ST-41/44 complex/Lineage 3	B	10	13-1
1	2973	ST-41/44 complex/Lineage 3	B	19	13-1
1	9218^N^	ST-41/44 complex/Lineage 3	B	19	13
3	9233^N^	ST-41/44 complex/Lineage 3	B	22	14-3
4	11	ST-11 complex/ET-37 complex	C	5-1	10-8
5	11	ST-11 complex/ET-37 complex	W135	5	2
1	9222^N^	ST-11 complex/ET-37 complex	W135	5	2
6	32	ST-32 complex/ET-5 complex	B	7-2	3
1	2493	ST-32 complex/ET-5 complex	B	7-2	3
1	3822	ST-32 complex/ET-5 complex	B	7–34	3
2	5138	ST-32 complex/ET-5 complex	B	7-2	3
1	9232^N^	ST-32 complex/ET-5 complex	B	7-2	3
1	9235^N^	ST-32 complex/ET-5 complex	B	7-2	3
2	183	ST-23 complex/Cluster A3	C	5-2	10-2
1	1161	ST-269 complex	B	22	9
1	9234^N^	ST-269 complex	B	18	25-1
2	9226^N^	ST-103 complex	29E	17	9
1	35	ST-35 complex	B	22-1	14
1	865	ST-865 complex	C	7	9
1	461	ST-461 complex	B	7-2	13-1
1	1624	ST-167 complex	Y	5-1	10-1
2	1768	Not assigned	Y	21	4
1	9221^N^	Not assigned	C	12-1	13-1
2	2003	Not assigned	B	17	9
1	9219^N^	Not assigned	Y	19-12	4
1	9220^N^	Not assigned	B	12-1	13-1

**^N^**New STs.

The 64 Isolates obtained in 2011 belonged to 24 ST and were grouped into five CC. A total of 22 (34.4%) isolates belonged to the ST-11 complex, of which 19 were serogroup W135, two were serogroup C and one was serogroup B. W135:ST-11 were isolated in 11 occasions, and there were two C:ST-11 isolates. W135:ST-1025 was detected in four cases and one B:ST-1025 was also seen. Two STs belonging to the ST-11 complex, ST-9911 and ST-9917, both serogroup W135, were identified here for the first time. Sixteen isolates belonged to the ST-41/44 complex, of which 13 were serogroup B, two W135 and one serogroup Y. ST-44 was the most prevalent with 12 isolates, 10 serogroup B, one W135 and one Y. ST-32 was also represented by 16 strains, all but one being serogroup B and nine of them being the central genotype ST-32. Two new STs were identified here, ST-9912 (one serogroup B and one W135) and ST-9916 (serogroup B). An additional five new STs were identified that are yet to be assigned to a clonal complex; three of them were serogroup C and two B.

### porA Typing

**Figure 2:**
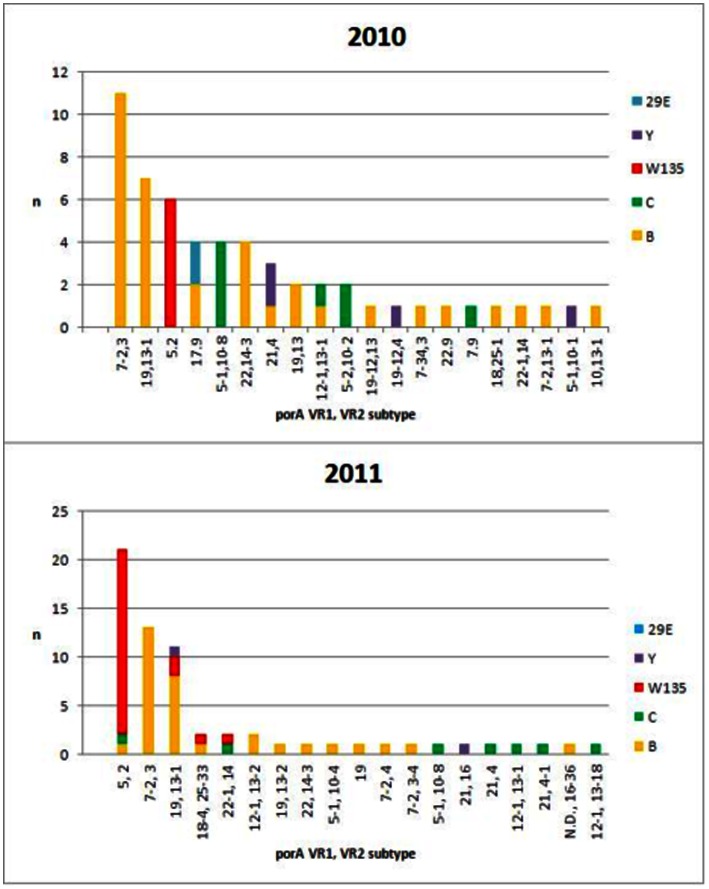
(a): Serosubtypes and serogroups identified during 2010. Each subtype is broken down into its constituent capsular groups. (b): Serosubtypes and serogroups identified during 2011. Each subtype is broken down into its constituent capsular groups.

*PorA* types were determined for all but one isolate. A total of 20 different VR1/VR2 combinations were found among the 55 isolates from 2010, 10 of which accounted for one isolate each (18,4% of isolates) (Table 2). Of the other 10 subtype combinations, 7 were found in up to 4 isolates (12,7%, collectively). The 3 major subtypes (24 isolates, 43.6%) were P1.7–2,3, and P1.19,13-1, which were found exclusively among serogroup B isolates, and P1.5,2, found exclusively among seogroup W135 isolates (Figure 2a). Serogroup C strains were mostly serosubtype P1.5-1,10-8.

In 2011, 19 different VR1/VR2 combinations were identified, of which 13 were identified only once (20,3%) (Table 3). P1.5,2, P17-2,3 and P1.19, 3-1 were also the most prevalent *porA* types, with 21 (32.8%), 13 (20.3%) and 11 (17.5%) isolates respectively. Serosubtypes P1.18-4, 25–33, P1.22-1, 14 and P1.12-1, 13-2 were identified twice each and together accounted for 9.3% of total isolates 2011 (Figure 2b).

**Table 3:** Genotypic characteristics of 64 isolates collected in 2011 causing meningococcal disease in Chile.

Number of isolates(N = 64)	ST	ST clonal complex	Serogroup	*porA* VR1	*porA* VR2
8	44	ST-41/44 complex/Lineage 3	B	19	13-1
1	44	ST-41/44 complex/Lineage 3	Y	19	13-1
1	44	ST-41/44 complex/Lineage 3	W135	19	13-1
1	44	ST-41/44 complex/Lineage 3	B	19	13-2
1	44	ST-41/44 complex/Lineage 3	B	22	14-3
1	3160	ST-41/44 complex/Lineage 3	W135	19	13-1
1	9909^N^	ST-41/44 complex/Lineage 3	B	5-1	10-4
1	5881	ST-41/44 complex/Lineage 3	B	19	13
1	41	ST-41/44 complex/Lineage 3	B	7-2	4
8	32	ST-32 complex/ET-5 complex	B	7-2	3
1	32	ST-32 complex/ET-5 complex	B	7-2	3-4
3	3822	ST-32 complex/ET-5 complex	B	7-2	3
1	9916^N^	ST-32 complex/ET-5 complex	B	7-2	3
1	2493	ST-32 complex/ET-5 complex	B	7-2	3
1	9912^N^	ST-32 complex/ET-5 complex	B	18-4	25–33
1	9912^N^	ST-32 complex/ET-5 complex	W135	18-4	25–33
11	11	ST-11 complex/ET-37 complex	W135	5	2
1	11	ST-11 complex/ET-37 complex	C	5	2
1	11	ST-11 complex/ET-37 complex	C	5-1	10-8
1	1025	ST-11 complex/ET-37 complex	B	5	2
4	1025	ST-11 complex/ET-37 complex	W135	5	2
1	5036	ST-11 complex/ET-37 complex	W135	5	2
1	9917^N^	ST-11 complex/ET-37 complex	W135	5	2
1	3298	ST-11 complex/ET-37 complex	W135	5	2
1	9911^N^	ST-11 complex/ET-37 complex	W135	5	2
1	35	ST-35 complex	W135	22-1	14
1	35	ST-35 complex	C	22-1	14
1	1466	ST-174 complex	Y	21	16
1	1768	Not assigned	C	21	4
1	9910^N^	Not assigned	C	12-1	13-1
1	9913^N^	Not assigned	C	21	4-1
1	9914^N^	Not assigned	B	N.D.	16–36
2	9915^N^	Not assigned	B	12-1	13-2
1	9220^N^	Not assigned	C	12-1	13–18

**^N^**:New STs.

**N.D.:** Not determined.

**Figure 3:**
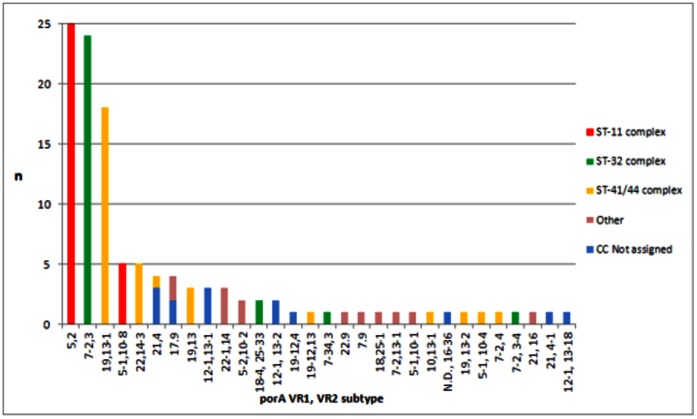
Serosubtypes and Clonal Complex identified during 2010 and 2011. Each subtype is broken down into its constituent capsular groups.

Figure 3 shows the distribution of VR1/VR2 combinations found among different clonal complexes combining all isolates from both years. W135:ST-11 complex isolates were exclusively associated with P1.5,2, whereas C:ST-11 complex presented the variant P15-1,10-8. Most ST-41/44 isolates were associated with a P1.19,13-1 *porA,* but other variants were also found among these isolates. ST-32 complex isolates presented mainly the variant P1.7-2,3 with some variants within the same family. A strain belonging to the newly identified ST-9914 presented an IS1301 inserted in the *porA* VR1 region and it was not possible to determine the *porA* variant.

## Discussion

This study represents, to the author’s knowledge, the first comprehensive investigation combining microbiological and molecular data on a collection of meningococcal strains from Chile for a two-year period. These isolates are fully representative of the epidemiology of the country in 2010–11, as meningococcal disease is of compulsory notification and the country has a very efficient epidemiological surveillance system that is well coordinated with the national reference laboratory in the ISP [17].

Our data show that three clonal complexes have predominated in Chile over the two years of the study: ST-11 complex, ST-41/44 complex and ST-32 complex, a distribution is similar to that described in Europe [25]. Serogroup B prevailed over the rest, with a total of 67 isolates mainly found in the ST-32 complex and ST-41/44, with 27 and 29 serogroup B isolates respectively, which is also consistent with what has been observed in Europe. However, whereas ST-11 complex isolates reported from Europe have been mainly associated with serogroup C [25], the vast majority of ST-11 complex strains isolated in Chile were serogroup W135, and only 6 C:ST-11 complex strains were detected in the study period, of which four were isolated in 2010. Moreover, a marked increase in the number of cases of IMD caused by the ST-11 complex serogroup W135 was detected in 2011 compared to the previous year, especially among patients under the age of 5. The W135:P1.5,2:ST-11 meningococcal strains found in Chile are indistinguishable from the one that was associated with an international outbreak among Hajj pilgrims in 2000 and 2001, suggesting that this phenomenon may be related to the global spread of this strain [9], [26]. In the light of the significant increase in the number of cases caused by this strain in Chile during 2012 [27], this is an important datum as it demonstrated that the strain was circulating in the country at least since 2010. Of the total 29 serogroup W135 strains found in our dataset, 26 belonged to the ST-11 complex, but ST-35, ST-32 and ST-41/44 complex serogroup W135 strains were also detected, highlighting the fact that it should not be assumed that all cases of IMD caused by this serogroup are due to the same meningococcal strain.

Other countries in Latin America have recently reported an increase of W135 invasive cases. Argentina has experienced an increase on W135 cases since 2008, and recently reported that nearly 49% of disease isolates characterized by their National Reference Laboratory in 2010 belonged to this serogroup. Brazil also reported an outbreak of disease caused by W135 in Southern provinces, in the border with Argentina [19], [28], [29]. In both countries the strain has been identified as the W135:P1.5,2:ST-11. Moreover, this Hajj 2000-related strain has been detected in other countries throughout the region (Ana Belén Ibarz-Pavón, personal communication). Molecular data from other Latin American countries experiencing disease caused by W135 strains would be of great value to understand the epidemiology and anticipate the occurrence of disease outbreaks. It is now more important than ever that Chile maintains a strong epidemiological and laboratory-based surveillance of IMD incorporating molecular typing, as a nationwide vaccination campaign with tetravalent conjugate vaccines targeting children under the age of five has been launched as a control measure for the recent increase in the number of cases caused by W135 [27], [30]. Molecular typing will allow the early detection of capsule switching and, more specifically, the early detection of the emergence of hyperinvasive strains.

An additional 11 serogroup B strains belonged to other CCs or were STs that were reported in this investigation for the first time, which further evidences the importance of generating molecular data, as association between CC and *porA* and *fetA* variants has been described [16], and it would be reasonable to assume that there might be variants circulating in Chile that have not yet been identified. Even though our data on *porA* does not suggest that this is the case, the number of strains processed is still small and limited to a short timespan compared to the amount of data that has been generated from other parts of the world. A strain belonging to the newly identified ST-9914 presented an IS1301 inserted in the *porA* VR1 region. This insertion element has been found in various regions across the *N.meningitidis* genome [31], including those related to capsule expression and transport [32], [33], as well as in the *porA* gene [34], [35], resulting on the non-expression of this protein. Both diversity of *porA* and inactivation of the protein expression could have important implications for the decision on a vaccination strategy with new upcoming universal vaccines that include this protein among their targets.

A total of 14 serogroup C strains were characterized in this study, eight from 2010 and six from 2011. Two of these strains were isolated from two siblings and identified as C:ST-183:ST-23 complex/Cluster A3, a complex that is usually associated with serogroup Y [36]. To date, only two ST-183 strains are reported to the PubMLST database, and both belong to serogroup Y. Moreover, the *porA* variant in one of the database strains is different to that found in our strains.

A total of 19 STs were identified in our dataset for the first time, 10 in 2010 and 9 in 2011, 11 of which were serogroup B and four serogroup C. ST-9220 was detected once in each year, but presenting a serogroup B capsule in 2010 and a C capsule in 2011. Two ST-9912 strains were detected in 2011: one B and one W135. The 29E:ST-9226 belonged to the ST-103 complex, and is therefore related to the C:ST-103 strain that caused a number of disease outbreaks in Brazil from 2004 onwards and prompted the introduction of the serogroup C conjugate vaccine (MenC) in their national immunization scheme [37]. Four new STs belonged to serogroup W135, of which three belonged to the ST-11 complex and presented the same *porA* variant as the hyperinvasive strain linked to the Hajj 2000 outbreak. These findings highlight the fact that meningococcal populations are highly dynamic and, consequently new potentially hyperinvasive strains are constantly appearing, advocating for the need of continuous monitoring to foresee their spread.

In conclusion, data from meningococcal isolates obtained from disease cases in Chile in 2010–2011 show that even though IMD rates in the country appears to be lower than those reported from Europe and the US prior to the implementation of conjugate vaccines in national immunization schemes, disease-causing strains are similar to those found circulating among other industrialized countries [25]. This work also shows that despite serogroup B being the most common among disease isolates, a marked increase in the number of cases caused by the Hajj 2000-related W135:ST-11 strain became apparent in 2011, and highlights the importance of maintaining a molecular characterization-based epidemiological surveillance in the country. These data demonstrate the necessity of implementing such characterization techniques across Latin America, as the early detection of the Hajj 2000-related strain could allow decision-making bodies to consider the implementation of prevention measures before or in the early stages of a potential outbreak. The detection of new STs among our strain collection further advocates for the generation of molecular data and particularly, characterization of OMPs included in the forecoming vaccine formulations. Finally, our work demonstrates the importance of maintaining and strengthening National Surveillance Programs integrating clinical, laboratory and epidemiological information, and updating guidelines on all these three components so data can be traced and compared to that generated all over the world.
